# Extending GroupStruct2: a Bayesian and machine-learning framework for testing taxonomic hypotheses using morphometric data

**DOI:** 10.3897/zookeys.1276.182331

**Published:** 2026-04-03

**Authors:** Kin Onn Chan, L. Lee Grismer

**Affiliations:** 1 Department of Integrative Biology, MSU Museum, Ecology, Evolution, and Behavior Program, Michigan State University, East Lansing, MI 48824, USA Department of Herpetology, San Diego Natural History Museum San Diego United States of America; 2 Herpetology Laboratory, Department of Biology, La Sierra University, 4500 Riverwalk Parkway, Riverside, CA 92505, USA Department of Biology, La Sierra University Riverside United States of America; 3 Department of Herpetology, San Diego Natural History Museum, P.O. Box 121390, San Diego, CA 92112, USA Department of Integrative Biology, MSU Museum, Ecology, Evolution, and Behavior Program, Michigan State University East Lansing United States of America; 4 El Serpentario y C.E.M.A de Baja California Sur, Brercha California e/ Nuevo Reforma y Guaycura, Fracc: Benito Juárez, CP 23090, La Paz, Baja California Sur, Mexico El Serpentario y C.E.M.A de Baja California Sur La Paz Mexico

**Keywords:** ANOVA, Boruta, DAPC, GroupStruct2, MFA, Morphology, PCA, *t*-test

## Abstract

Despite considerable advances in statistical methods, taxonomic delimitation using morphometric data (morphometric delimitation) has not significantly progressed beyond the use of simple summary statistics or univariate tests to quantify differences among predefined operational taxonomic units (OTUs). These methods typically rely on visual inspection of graphs or *p*-value thresholds to determine if character means are statistically different. Tiburtini et al. (2025) introduced a conceptually different approach for morphometric delimitation using Bayesian model-testing and Gaussian Mixture Models (GMM). This approach can infer morphological clusters with or without *a priori* OTU groupings and jointly evaluates the fit of alternate taxonomic hypotheses to the data, providing a probabilistic, model-based framework that moves beyond traditional significance testing. Additionally, a machine-learning method was proposed to identify diagnostic characters based on a Random Forest classification algorithm. Initially developed for plant morphometrics, we adapted Tiburtini et al.’s approach for any morphometric dataset and integrated it into GroupStruct2, a Shiny R-based application with a full graphical user interface that also includes conventional statistical methods (e.g. univariate/multivariate tests, PCA, DAPC, MFA). We demonstrate that a more robust, nuanced, and comprehensive perspective on morphological variation and character diagnoses can be achieved using GroupStruct2’s integrative workflow that combines classical statistical analyses with Bayesian GMM and machine-learning methods. The integration of frequentist and Bayesian methods within a user-friendly graphical interface democratizes access to robust statistical analyses and enables researchers to adopt quantitative rigor in taxonomic studies.

## Introduction

For decades, morphometric data (continuous measurements of morphological traits) have been used to quantify and characterize differences among organisms (Blackith and Reyment 1971). Even in the genetic era, morphometric data remain central to systematic biology, largely because morphological variation contains important diagnostic information and morphometric data can be collected without specialized equipment or highly technical skills. In specimen-based systematics and taxonomy, morphometric data remain a vital source of evidence for species delimitation—the process of inferring species boundaries and assessing species distinctiveness (Sites et al. 2003).

We emphasize an important conceptual distinction between morphometric and species delimitation. Morphometric delimitation refers to the process of characterizing morphological group-structure based solely on morphometric data, while species delimitation is the broader integrative framework that synthesizes multiple independent lines of evidence (e.g. morphology, genetics, ecology, biogeography, behavior) to infer evolutionary lineages and delimit species boundaries (Padial et al. 2010). Morphometric delimitation represents only a single line of supporting evidence within the broader species delimitation framework and cannot, by itself, establish species boundaries because morphological structure can arise from factors other than lineage divergence (e.g. propinquity of relationship, population structure, polymorphism, geographic variation, etc.). Although performing multiple morphometric analyses strengthens results, the results are nevertheless based on the same data, are not independent, and collectively represent a single source of information or evidence and may not necessarily be congruent with phylogenetic-based delimitation.

Morphometric delimitation is rooted in numerical taxonomy, where multiple traits are measured and analyzed using various mathematical algorithms with the primary aim of grouping similar organisms and differentiating dissimilar groups (Michener and Sokal 1957; Sokal and Michener 1958). Although the field has expanded from traditional linear measurements (e.g. snout–vent length, head width, femur length, etc.) to include landmark-based geometric morphometrics (Rohlf and Marcus 1993), the overall approach for morphometric delimitation within the context of systematics and taxonomy has remained relatively unchanged: morphometric data for multiple characters are collected from operational taxonomic units (OTUs) and analyzed to quantify differences among them. This traditional framework typically assesses OTU distinctiveness through visual inspection of OTU separation in morphospace, discriminant analysis, and null hypothesis significance testing (NHST) to determine if observed character differences are statistically significant.

Null hypothesis significance testing (NHST) approaches have dominated morphometric delimitation (Chan and Grismer 2021), and while they can effectively characterize morphological differences among pre-defined groups, they have well-documented limitations (Halsey 2019). First, the widespread reliance on subjective *p*-value significance thresholds (typically α = 0.05) creates a false dichotomy between “significant” and “non-significant” results. This binary framework can inadvertently encourage researchers to focus on achieving statistical significance rather than evaluating the biological meaning of morphological differences, thereby devaluing legitimate interpretations (Nakagawa and Cuthill 2007) and eliminating meaningful results. Second, *p*-values are frequently misinterpreted: a *p* < 0.05 does not indicate that OTUs are truly distinct, nor does it quantify the strength of evidence for distinctiveness—it merely indicates that the observed differences would be unlikely under a specific null hypothesis. Moreover, with sufficiently large sample sizes, even trivial morphological differences can become statistically significant. Third, traditional approaches typically test each comparison independently (e.g. pairwise comparisons between OTUs), without a coherent framework for jointly evaluating multiple alternative taxonomic hypotheses that differ in the number or composition of OTUs.

In contrast, a fundamentally different approach was developed for molecular “species” delimitation, where alternate taxonomic hypotheses (models) are explicitly formulated and evaluated within a Bayesian framework to determine how well each model fits the genetic data, allowing direct comparisons of competing taxonomic scenarios (Leaché et al. 2014; Kornai et al. 2024). This model-based Bayesian framework represents a significant conceptual shift from NHST approaches by offering probabilistic inferences that better align with the often uncertain and continuous nature of morphological variation, and by extension, taxonomic decisions. Rather than dichotomous significance cut-offs, Bayesian methods yield posterior probabilities that directly quantify the degree of support and uncertainty for competing hypotheses. Bayesian metrics such as Bayes Factors enable joint evaluation of multiple plausible taxonomic scenarios, formally quantifying the relative strength of evidence for each competing hypothesis without arbitrary significance thresholds (Jeffreys 1935; Kass and Raftery 1995). This framework has been widely adopted for molecular data (Grummer et al. 2014; Leaché et al. 2014; Smith and Carstens 2022) but remained absent from morphometric approaches until recently.

Tiburtini et al. (2025) introduced a Bayesian model-based approach for morphometric delimitation in plants based on Gaussian Mixture Models (GMM), and a machine-learning algorithm to identify diagnostic characters. Inspired by their work, we developed a generalized framework that applies to any morphometric dataset and integrated it into a broader workflow that combines classical NHST methods with their proposed Bayesian GMM and machine-learning approach. We implemented this integrative workflow in GroupStruct2, a Shiny R application with a complete graphical user interface (GUI) that requires no coding expertise (Chan and Grismer 2025), making robust morphometric analyses accessible to the broader systematic biology community across all taxonomic groups. Here, we demonstrate how fundamentally different approaches can provide complementary lines of evidence to yield a more nuanced, in-depth, and holistic perspective on species boundaries and the evolution of morphometric variation.

## Methods

We adapted the original Bayesian GMM and machine-learning code from Tiburtini et al. (2025) and incorporated it into GroupStruct2, a fully GUI-based Shiny R application for comprehensive morphological analysis and data visualization (Chan and Grismer 2025). GroupStruct2 requires either R (R Core Team 2025) or RStudio (Posit Team 2025) and can be downloaded and launched using only 3 or 4 lines of code:

install.packages(“devtools”)

devtools::install_github(“chankinonn/GroupStruct2”)

library(GroupStruct2)

groupstruct2()

The first line installs the devtools package that enables the installation of R packages from GitHub. The second line uses devtools to install GroupStruct2. The third line loads GroupStruct2, and the fourth line launches the application. All code is freely available at https://github.com/chankinonn/GroupStruct2.

Based on the type of morphological data, GroupStruct2 provides several analytical workflows arranged into three main modules for (1) Meristic, (2) Morphometric, and (3) Mixed data (meristic + morphometric + categorical). Within each main module are submodules for various analyses and data visualization. The Bayesian GMM analyses are only applicable to continuous morphometric data and are therefore only available within the Morphometric module under the Morphometric Delimitation submodule. Four types of analyses are implemented in the Morphometric Delimitation submodule:

### Unsupervised clustering

This analysis uses GMM implemented in the mclust R package (Scrucca et al. 2016) to infer the number of morphological clusters (G) without relying on *a priori* OTU labels. The algorithm assumes that morphometric data are generated from a mixture of underlying multivariate Gaussian (normal) character data distributions, where each cluster represents one component of the mixture. For each potential number of clusters (G = 1 to user-defined maximum), the algorithm fits multiple models differing in covariance structure that defines the geometric properties of clusters, including volume, shape, and orientation. The Bayesian Information Criterion (BIC) balances model fit against complexity to select the optimal number of clusters and corresponding covariance structure (Schwarz 1978). By cross-referencing inferred cluster assignments to the original OTU labels, this analysis reveals whether morphological variation naturally separates into discrete groups and how accurately cluster memberships match taxonomic designations. Three patterns may emerge: (1) perfect correspondence—each cluster contains only one OTU, indicating strong morphometric differentiation aligning with *a priori* OTU groupings; (2) mixed clusters—multiple OTUs within clusters, suggesting morphological overlap, similarity, or cryptic species, suggesting taxonomic re-evaluation; or (3) split OTUs—single OTUs distributed across multiple clusters, indicating potential cryptic diversity, misidentification, or intraspecific variation. This method would be especially useful in hypothetical species delimitations in the absence of a phylogeny.

### Supervised clustering

In contrast to unsupervised clustering that seeks to identify clusters without relying on *a priori* OTU labels, supervised clustering uses pre-defined OTU groupings (determined by the user) to train discriminant models and evaluates alternative delimitation schemes through a stepwise merging algorithm. This provides a data-driven, quantitative approach to test whether OTUs should be lumped or maintained as separate. Starting with the original user-defined OTU grouping, the algorithm employs EDDA (Eigenvalue Decomposition Discriminant Analysis) models, which allow each group to have its own covariance structure, and iteratively tests all pairwise merges at each step. At each iteration, BIC is calculated for every possible pairwise combination, and the merge yielding the highest BIC is selected. Note that the mclust function calculates BIC = 2(log-likelihood – penalty), which maximizes BIC for the best model (Scrucca et al. 2016). This process continues until all OTUs are merged into a single group, generating a hierarchical sequence of delimitation schemes from maximum splitting (all original OTUs separate) to maximum lumping (all OTUs combined). The Bayes Factor metric is used to quantify the strength of evidence for each scheme relative to the best-supported model, and posterior model probabilities indicate the relative likelihood of each delimitation given the data. This greedy stepwise approach follows a single optimal merging path rather than exhaustively testing all possible partitions, which would be computationally prohibitive (e.g. beyond ~10 OTUs). For comprehensive testing of specific taxonomic hypotheses, users should employ the Bayesian hypothesis-testing framework (see below). For best results with supervised clustering, users should start with the most “splitty” scheme plausible, as the algorithm can only merge groups, not split them.

### Hypothesis testing

While supervised clustering explores all possible merges based on the initial pre-defined OTU grouping, the hypothesis-testing analysis allows researchers to explicitly define and compare specific taxonomic schemes informed by prior analyses or external evidence (e.g. genetics, geography, ecology). Similar to supervised clustering, this analysis fits GMMs under different EDDA covariance parameterizations for each hypothesis and computes BIC, Bayes factors, and posterior model probabilities assuming a flat prior (equal prior probability for all hypotheses). This approach provides more flexibility and focused testing of biologically plausible scenarios without exhaustively testing all merging combinations, many of which may be implausible, as would be the case with supervised clustering. Critically, unlike supervised clustering, which can only merge groups, this analysis can evaluate splits within OTUs to test for cryptic diversity.

### Diagnostic character identification

Identifying diagnostic morphological characters is essential for taxonomic descriptions and identification keys. This analysis uses the Boruta R package (Kursa and Rudnicki 2010), which implements a machine-learning feature selection algorithm built on Random Forest to identify variables that are statistically important for distinguishing groups. The algorithm works by creating randomized “shadow” copies of all variables (= morphometric characters) and iteratively comparing the importance of real characters against these shadows. Character importance is measured by how much classification accuracy drops when a character’s values across groups are scrambled: characters causing large accuracy drops contain important information needed to distinguish groups. Boruta uses permutation-based statistical tests to classify characters as Confirmed (significantly more important than the best shadow variable—these are diagnostic characters), Rejected (importance no better than random noise), or Tentative (uncertain, may require more iterations). It is important to note that this analysis employs a frequentist NHST approach (*p*-values) to assess significance instead of a Bayesian framework. The analysis identifies which characters best discriminate OTUs, not how different OTUs are. For best results, this analysis should be accompanied by univariate tests to provide statistical significance for individual trait differences.

**Figure 1.**
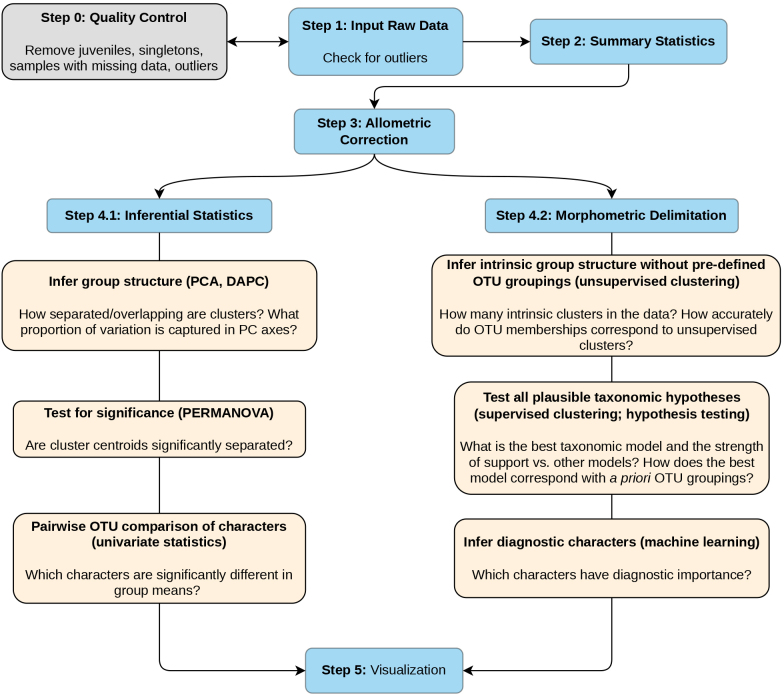
An example workflow for morphometric delimitation using GroupStruct2. Step 0 (grey box) is not part of GroupStruct2 and should be performed independently before using the application. Steps 1–5 (blue boxes) represent submodules within the main Morphometric module. Each submodule contains a suite of stand-alone analyses that can be performed to answer different questions (tan boxes). Analyses in the Inferential Statistics (Step 4.1) and Morphometric Delimitation (Step 4.2) submodules are based on fundamentally different conceptual frameworks, and the results of each should be compared to obtain more holistic taxonomic conclusions. Bayesian GMM and machine-learning analyses introduced by Tiburtini et al. (2025) and this study are implemented in the Morphometric Delimitation (Step 4.2) submodule.

Below, we provide an example workflow demonstrating how NHST and Bayesian methods can be integrated for morphometric delimitation in GroupStruct2. Steps 1–5 correspond to submodules within the Morphometric module (Fig. 1). Additional documentation for each analysis is provided within the GroupStruct2 interface.

### Example workflow

***Step 0 (Data Quality Control)***—Before using GroupStruct2, raw data need to be curated by removing missing data, singletons (OTUs represented by only one specimen), juveniles, and erroneous data. If sexual dimorphism is present, males and females should be separated into different datasets and analyzed separately.

***Step 1 (Input Data)***—After initial data quality control, use the Input Data submodule to upload the raw data. Detailed instructions on how to format the input data are provided within the application. The Input Data submodule also contains an outlier test to check for potentially erroneous values. Users are also encouraged to examine scatter, box, and violin plots in the Visualization submodule (Step 5) to check for erroneous data.

***Step 2 (Summary Statistics)***—This submodule generates a formatted summary statistics table (N; Mean ± SD; Min—Max) that can be downloaded as a publication-ready table.

***Step 3 (Allometric Correction)***—Raw measurements need to be adjusted to remove proportional variation relating to body size. This submodule uses the Thorpe equation (Thorpe 1975) and is adapted from the original GroupStruct R package (Chan and Grismer 2022). For organisms that do not exhibit allometric growth, this analysis can be skipped by selecting the “No Correction” option. Any changes made in this submodule will be reactively transferred to downstream analyses; therefore, users are encouraged to explore how allometric correction (or the lack thereof) affects downstream analyses.

***Step 4.1 (Inferential Statistics) and Step 4.2 (Morphometric Delimitation)***—The Inferential Statistics submodule provides NHST-based analyses, while the Morphometric Delimitation submodule implements Bayesian GMM and machine-learning approaches for hypothesis testing and diagnostic character identification. The suggested workflow integrates both frameworks:

#### Exploratory analysis

Perform PCA and DAPC to visualize group structure and quantify character contributions to overall variation. Follow with a PERMANOVA on PCA scores to test whether group separation in multivariate space is statistically significant.

Conduct unsupervised clustering to infer the number of morphological clusters without relying on pre-defined OTU labels. Compare the inferred number of clusters with the number of OTUs from the initial input data.

Examine the correspondence table and “PCA Clusters” plot (Visualization module) to assess cluster-OTU agreement. Mixed clusters indicate morphological overlap between OTUs, while OTUs split across multiple clusters suggest either substantial intraspecific variation, misidentification, or potential cryptic diversity.

#### Hypothesis testing

Use supervised clustering to evaluate a stepwise sequence of lumping scenarios. The algorithm begins with the original OTU assignments and iteratively merges the two most similar groups at each step based on BIC, continuing until all OTUs are lumped into a single cluster. This exploratory approach identifies which groups are most morphologically similar.

Alternatively, use the Hypothesis testing analysis to rigorously compare specific, user-defined taxonomic schemes (uploaded as a separate file). This allows exhaustive testing of any plausible delimitation hypotheses. Both supervised clustering and hypothesis testing use the same GMM framework and will yield identical results if the hypotheses compared are the same.

#### Diagnostic character identification

Apply the machine-learning analysis to identify which morphometric variables best discriminate among OTUs. Users can select specific groups to compare, and results can be visualized as ridge or box plots in the Visualization module.

Complement these results with univariate tests (Inferential Statistics module) to determine whether diagnostic characters show statistically significant differences between groups. Note that characters may be diagnostic (high discriminatory power) without being significantly different in magnitude, and vice versa.

Analyses in these submodules are independent and do not need to be performed in any particular order. Therefore, the workflow outlined here is merely a suggestion, and users are encouraged to explore each analysis in detail.

***Step 5 (Visualization)***—All graphical plots can be viewed and downloaded in this submodule. Plots are based on the ggplot2 architecture (Wickham 2016) and are highly customizable to tailor to the specific needs of each researcher. The quality (size) and format (JPEG or PDF) of each plot can be specified to produce publication-ready figures.

We demonstrate how to perform morphometric delimitation using the GroupStruct2 workflow described above using a published empirical dataset of 17 morphometric characters from three species of dusky salamanders from the southeastern United States: *Desmognathus conanti*, *D. valentinei*, and *D. pascagoula*—*Desmognathus pascagoula* was recently split from *D. valentinei* based on morphological and genetic data (Pyron et al. 2023). Briefly, we performed allometric size correction followed by PCA and DAPC. To determine whether the centroids of PCA clusters were significantly different, we employed PERMANOVA with 50,000 iterations. We then implemented the GMM Bayesian hypothesis-testing framework to evaluate the distinction between the three species using both unsupervised and supervised clustering. Finally, we used the Boruta machine-learning algorithm to identify diagnostic characters between pairs of taxa, supplemented by univariate *t*-tests to determine significant differences in character means. Character abbreviations and definitions follow Pyron et al. (2023) and are provided in Suppl. material 1: table SS1. All tables and figures were generated directly from GroupStruct2 with minimal post-processing.

## Results

**Figure 2.**
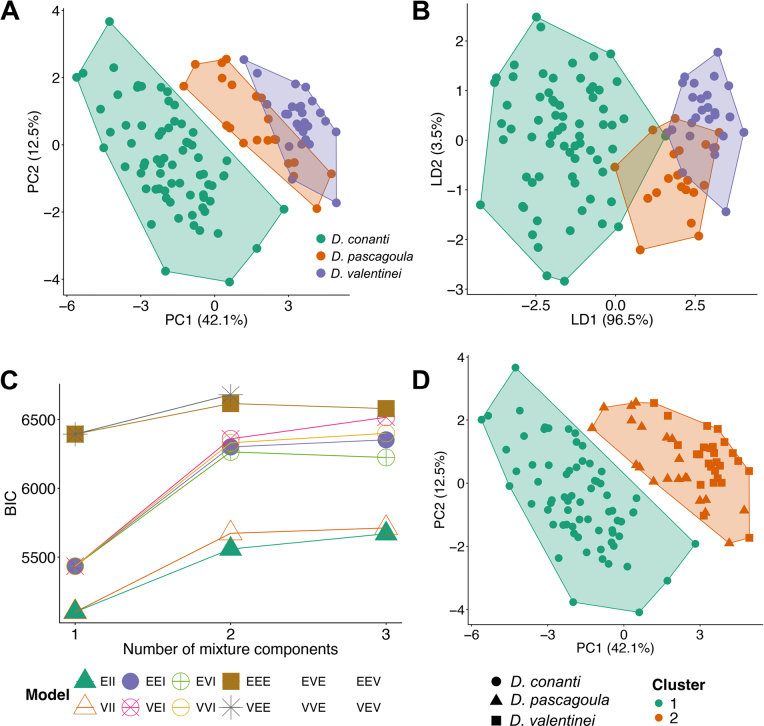
PCA (**A**) and DAPC (**B**) plots outlined with convex hulls, respectively; **C**. BIC scores (y-axis) and number of inferred clusters (x-axis) from the unsupervised clustering analysis where colors and symbols represent the different models tested (see Suppl. material 1: table SS2 for model definitions); **D**. Results of the supervised clustering analysis visualized on a PCA plot to show the congruence of cluster memberships to the original species groupings. Inferred clusters are colored and bounded by convex hulls, while species groupings are represented by symbols (circles, squares, and triangles).

PCA and DAPC plots showed clear separation between *Desmognathus conanti* and *D. valentinei* along PC1 and LD1, respectively. Less separation was observed between *D. pascagoula* and *D. valentinei* (Fig. 2). The PERMANOVA analysis based on PCA scores showed that all pairwise species comparisons were significantly different (Table 1). The strongest divergence was observed between *D. conanti* and *D. valentinei* (*R*^2^ = 0.33), indicating substantial separation in morphospace. Comparisons between *D. conanti* and *D. pascagoula* (*R*^2^ = 0.18) and between *D. pascagoula* and *D. valentinei* (*R*^2^ = 0.17) showed more moderate levels of differentiation. Although the *D. conanti* comparisons produced larger *F*-statistics, this likely reflects the larger sample size for this species rather than a substantially greater effect size.

**Table 1.** Results from the PERMANOVA analysis testing the significance of OTU separation based on PCA scores.

Taxa comparison	*F*-statistic	*R* ^2^	*p*-value	*p*-adjusted
*D. conanti* vs *D. pascagoula*	19.128301	0.178555	0.000020	0.000060
*D. conanti* vs *D. valentinei*	45.473795	0.328393	0.000020	0.000060
*D. pascagoula* vs *D. valentinei*	9.103686	0.168264	0.000040	0.000120

Unsupervised GMM clustering inferred two clusters and the VEE model as the most optimal fit (Fig. 2C; see Suppl. material 1: tables S2, S3 for more results and a detailed explanation of model covariance structures). Cluster 1 included all *D. conanti* samples, while Cluster 2 comprised *D. pascagoula* + *D. valentinei* (Fig. 2D; Table 2). By evaluating all possible merging schemes based on the original species groupings, the supervised clustering analysis also inferred a two-species model as the best fit to the data by lumping *D. pascagoula* and *D. valentinei* (Table 3). The second-best model separated all three species but there was relatively strong support against it (∆BIC = 11).

**Table 2.** Correspondence table from the unsupervised clustering analysis showing memberships of the two inferred clusters. Columns = clusters; rows = number of specimens belonging to a particular cluster.

Cluster	1	2
*D. conanti*	69	0
*D. pascagoula*	0	21
*D. valentinei*	0	26

**Table 3.** Results from the supervised clustering analysis. K = number of clusters; BIC = Bayesian Information Criterion; BF = Bayes Factor; PP = posterior probability.

Taxonomic hypotheses	K	BIC	∆BIC	BF	PP
*D. conanti* | *D. pascagoula*-*D. valentinei*	2	6681	0	1	0.996
*D. conanti* | *D. pascagoula* | *D. valentinei*	3	6670	11	244.945	0.004
*D. conanti*-*D. pascagoula*-*D. valentinei*	1	6394	287	2.107E+62	0

**Figure 3.**
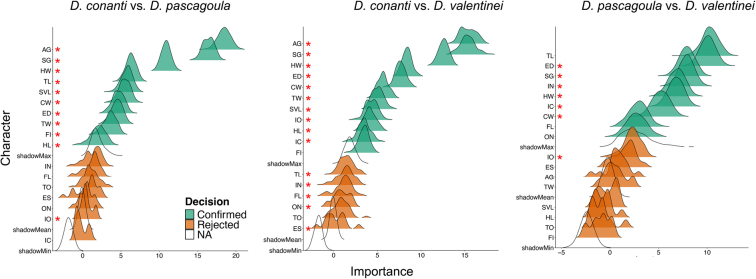
Ridge plots from the Boruta machine-learning analysis to identify diagnostic characters between pairwise species comparisons. Characters that were significantly different (*p* < 0.05) in univariate *t*-tests are shown as red asterisks (*) next to the corresponding character. See Suppl. material 1: table SS1 for character definitions.

In general, most diagnostic characters identified by the Boruta machine-learning analysis were also significantly different in NHST tests (Fig. 3). However, there were discrepancies where several confirmed characters from the Boruta analysis were not significantly different in *t*-tests and vice versa.

## Discussion

Our results demonstrate that integrating NHST with Bayesian and machine-learning frameworks provides a more nuanced characterization of morphological variation than any single approach. For example, while PCA and PERMANOVA inferred significant separation among all three species (Table 1), the Bayesian GMM analyses favored a two-species model. Specifically, the top four models from unsupervised clustering lumped *Desmognathus pascagoula* and *D. valentinei* into a single morphological cluster (Suppl. material 1: table SS2). This result was also corroborated by supervised clustering (Table 3). However, the distinctiveness of *D. pascagoula* and *D. valentinei* is validated by independent genetic and geographic data (Pyron et al. 2023). This discordance underscores why morphometric data alone are insufficient for species delimitation and are better suited for generating, rather than validating taxonomic hypotheses. Furthermore, the machine-learning approach revealed that diagnostic utility and statistical significance are not synonymous. Random Forest identified several characters with high importance for classification that lacked statistical significance in univariate tests (Fig. 3), illustrating the value of complementing classical hypothesis testing with predictive modeling approaches.

The GroupStruct2 workflow demonstrates that no single analytical method is inherently superior, and optimal results emerge from their cohesive integration (Padial et al. 2010). However, even comprehensive statistical analyses cannot overcome fundamental data limitations. GMMs assume each OTU follows a multivariate Gaussian (normal) distribution, and violation of this assumption could produce erroneous results. This normality requirement also precludes the analysis of non-Gaussian morphological data types such as meristic counts or categorical characters, though multiple factor analysis (MFA) can integrate such data when available (Grismer 2025). Bayesian methods also introduce additional considerations. GroupStruct2 implements flat priors, treating all taxonomic hypotheses as equally probable *a priori*, which may not reflect biological reality. Moreover, BIC scores evaluate relative model fit, not absolute correctness (i.e. it may choose the least wrong model). Similarly, Bayes Factor thresholds (e.g. BF > 20 for “strong evidence”) provide interpretive guidelines but remain somewhat subjective.

Given the complementary strengths and limitations of frequentist and Bayesian approaches, neither method alone provides sufficient evidence for taxonomic decisions, especially in the absence of phylogeny. Frequentist methods (univariate tests, PERMANOVA) provide hypothesis testing frameworks and effect size estimates that quantify the magnitude of trait differences between groups. In contrast, Bayesian GMM methods evaluate relative support for competing taxonomic models through model comparison, offering a probabilistic framework that can reduce subjectivity in taxonomic decision making. Critically, high statistical support does not equate to taxonomic validity. These methods identify morphological structure in the data but cannot distinguish whether that structure reflects lineage independence, population structure, geographic variation, phenotypic plasticity, or polymorphism. Morphometric analyses detect patterns—they do not diagnose evolutionary processes. Therefore, regardless of statistical rigor or support, these methods identify morphological clusters, not evolutionary lineages. Consequently, morphometric evidence must be integrated with independent data sources that provide biological context for interpreting morphological patterns.

Despite the availability of robust statistical methods, their adoption in taxonomic research has been limited by technical barriers requiring programming expertise or familiarity with multiple software platforms. GroupStruct2 addresses these obstacles by consolidating analytical tools into an accessible graphical interface requiring no coding experience. Results can be downloaded in tabular formats, and visualizations are customizable for publication-quality figures. By democratizing access to quantitative methods, GroupStruct2 aims to bridge statistical methodology and taxonomic practice, empowering researchers to leverage these approaches as hypothesis-generating tools within integrative taxonomy.

## Additional information

### Use of AI

The following AI tools were used in the preparation of this manuscript:

**Description**: Claude.ai was used to check and improve the writing.

### Author ORCIDs

Kin Onn Chan https://orcid.org/0000-0001-6270-0983

L. Lee Grismer https://orcid.org/0000-0001-8422-3698

### Data availability

All of the data that support the findings of this study are available in the main text or Supplementary Information.

## Supplementary materials

Supplementary material 1 Supplementary tables Data type pdf Explanation note **table SS1**. List of characters used in this study. **table SS2**. Top 20 models from the unsupervised clustering analysis. **table SS3**. Covariance structure parameterizations for Gaussian Mixture Models evaluated in the unsupervised clustering analysis. https://binary.pensoft.net/file/1582237This dataset is made available under the Open Database License (http://opendatacommons.org/licenses/odbl/1.0/). The Open Database License (ODbL) is a license agreement intended to allow users to freely share, modify, and use this Dataset while maintaining this same freedom for others, provided that the original source and author(s) are credited. Kin Onn Chan, L. Lee Grismer
