# Supplementary material for: Extending GroupStruct2: a Bayesian and machine-learning framework for testing taxonomic hypotheses using morphometric data
Source: Zookeys. 2026 Apr 3;1276:125–38. doi: 10.3897/zookeys.1276.182331 (PMC13069391; doi:10.3897/zookeys.1276.182331)
Supplement: Supplementary material 1 — Supplementary tables [file zookeys-1276-125_article-182331__-s001.pdf]

Supplementary Material

Testing Taxonomic Hypotheses and Identifying Diagnostic Characters: A Bayesian and Machine Learning Approach

Kin Onn Chan & L. Lee Grismer

Table S1. List of characters used in this study.

Character Abbreviation	Definition
SVL	Snout-vent-length
TL	Tail length
AG	Axilla-groin length
CW	Cchest width
FL	Femur length
HL	Humerus length
SG	Snout-gular length
TW	Tail width
TO	Length of third toe
FI	Length of third finger
HW	Head width
ED	Eye diameter
IN	Internarial distance
ES	Eye-snout distance
ON	Orbito-narial distance
IO	Inter-orbital distance
IC	Inter-canthal distance

Table S2. Top 20 models from the unsupervised clustering analysis. G = number of clusters; BIC = Bayesian Information Criterion.

Rank	G	Model	BIC	Delta_BIC
1	2	VEE	6680.78626	0
2	2	VVE	6649.62172	31.1645357
3	2	EEE	6615.98716	64.7990909
4	2	EVE	6595.43857	85.3476866
5	3	EEE	6579.30506	101.481194
6	3	VEI	6516.1959	164.590359
7	3	VVI	6399.69383	281.092425
8	1	EEE	6393.77516	287.011093
9	1	VEE	6393.77516	287.011093
10	1	EVE	6393.77516	287.011093
11	1	VVE	6393.77516	287.011093
12	1	EEV	6393.77516	287.011093
13	1	VEV	6393.77516	287.011093
14	1	EVV	6393.77516	287.011093
15	1	VVV	6393.77516	287.011093
16	2	VEI	6359.52555	321.260702
17	3	EEI	6352.54931	328.236941
18	2	VVI	6330.72668	350.059574
19	2	EEI	6300.66317	380.123086
20	2	EVI	6264.50292	416.283333

Table S3. Covariance structure parameterizations for Gaussian Mixture Models evaluated in the unsupervised clustering analysis. E = equal across clusters, V = variable across clusters, I = identity matrix. Volume = cluster size, Shape = geometric shape of clusters, Orientation = principal component directions. Spherical models assume equal variance in all directions; diagonal models allow variable variance along coordinate axes; ellipsoidal models allow full covariance structure with arbitrary orientation.

Model	Volume	Shape	Orientation	Distribution
EII	Equal	Equal	NA	Spherical
VII	Variable	Equal	NA	Spherical
EEI	Equal	Equal	Coordinate axes	Diagonal
VEI	Variable	Equal	Coordinate axes	Diagonal
EVI	Equal	Variable	Coordinate axes	Diagonal
VVI	Variable	Variable	Coordinate axes	Diagonal
EEE	Equal	Equal	Equal	Ellipsoidal
EVE	Equal	Variable	Equal	Ellipsoidal
VEE	Variable	Equal	Equal	Ellipsoidal
VVE	Variable	Variable	Equal	Ellipsoidal
EEV	Equal	Equal	Variable	Ellipsoidal
VEV	Variable	Equal	Variable	Ellipsoidal
EVV	Equal	Variable	Variable	Ellipsoidal
VVV	Variable	Variable	Variable	Ellipsoidal
